# Patient and public involvement and engagement in clinical trials at scale: Analysis of the first 3250 responses on the POrtal for Patient and Public Engagement in Dementia (POPPED)

**DOI:** 10.1002/alz.71113

**Published:** 2026-02-28

**Authors:** Hongyi Qin, Linda Pointon, James Carpenter, Vanessa Raymont, Ross Dunne, Suzanne Reeves, Shabinah Ali, Sabahat Iqbal, Cristina Bonet‐Olivares, Joanne Whittle, Laura Rizzo, Paresh Malhotra, Benjamin R Underwood, the AD‐SMART team

**Affiliations:** ^1^ Department of Public Information Resources Management Zhejiang University Hangzhou China; ^2^ Department of Psychiatry University of Cambridge Cambridge UK; ^3^ MRC Clinical Trials Unit University College London London UK; ^4^ Department of Psychiatry University of Oxford Oxford UK; ^5^ Department of old age psychiatry Greater Manchester Mental Health NHS Foundation Trust Manchester UK; ^6^ Department of psychiatry University of Manchester Manchester UK; ^7^ Department of old age psychiatry University College London London UK; ^8^ Department of neurology Imperial College London London UK; ^9^ UK Dementia Research Institute Care Research and Technology Centre Imperial College London London UK; ^10^ Cambridgeshire and Peterborough NHS Trust Cambridge UK

**Keywords:** Alzheimer's disease, clinical trial, discrete choice experiments (DCEs), drug preferences, patient and public involvement and engagement (PPIE)

## Abstract

**INTRODUCTION:**

Patient and public involvement and engagement (PPIE) improves research quality but is often limited in scale. This study explored the potential for large‐scale PPIE using a Web‐based approach.

**METHODS:**

We created an online portal to gather public views on dementia research and a UK‐based adaptive platform trial testing repurposed Alzheimer's disease drugs. Participants ranked four anonymized drugs and completed discrete choice experiments on treatment trade‐offs. Analyses were stratified by sex, age, and dementia experience.

**RESULTS:**

Among 3250 people across 27 countries (87.4% UK‐based), 79.6% expressed positive attitudes toward the trial. Metformin was the most preferred drug, followed by atomoxetine, isosorbide mononitrate, and levetiracetam. Probability of severe side effects was the most influential treatment attribute, followed by probability of mild side effects and type of evidence. Subgroup analyses supported the main findings.

**DISCUSSION:**

Web‐based PPIE can effectively inform dementia research at scale and provides a reusable resource for other studies.

## Highlights

- Described a Web‐based platform (POrtal for the Patient and Public Engagement in Dementia Research [POPPED]) to scale patient and public involvement and engagement (PPIE) in dementia research.
- Engaged 3000+ participants globally to prioritize Alzheimer's disease (AD) drugs for the Alzheimer's Disease‐Systematic Multi‐Arm Adaptive Randomized Trial (AD‐SMART) trial.
- Discrete choice experiments (DCEs) found side effects outweighed all other drug attributes.
- Built a 2000‐person cohort for future dementia PPIE activities.

## 1 BACKGROUND

Patient and public involvement and engagement in research (PPIE) is a process by which patients and the public are active partners in all stage of the research cycle, from setting priorities and research questions, to design and dissemination of findings.^1^, ^2^ Standards exist for PPIE based on principles of inclusivity, support, working together, governance, communication and impact.^3^, ^4^ Further guidance exists on measuring the impact of PPIE and publishing findings.^5^, ^6^

Despite this progress, there is still considerable room for development.^7^ First, many PPIE groups are often small in number and may not include people from underserved groups.^8^ This limitation is particularly concerning in dementia research, where an estimated 60 million people worldwide living with the condition and increasing numbers of families and caregivers are affected. In some cases, PPIE is limited, focusing on dissemination rather than involving collaborators in a more integrated and participatory role.^9^, ^10^ More meaningful engagement would include working with PPIE contributors throughout the research process from initial designing to implementation.^11^ In other areas of medicine, researchers have attempted to address these limitations by engaging online with a global community.^12^ However, whether web‐based approaches can achieve scalable, inclusive PPIE in dementia research remains unknown. To address this gap, we investigated the potential for engaging with patients and the public at scale using a novel purpose‐built web‐based portal (POrtal for the Patient and Public Engagement in Dementia Research [POPPED]) to facilitate mass feedback.

This study was part of a wider initiative to design and implement a UK‐wide platform in clinical Alzheimer's Disease (Alzheimer's Disease‐Systematic Multi‐Arm Adaptive Randomized Trial [AD‐SMART]) designed in early 2025. A key consideration for AD‐SMART is which candidate drugs should be prioritized for initial evaluation in the platform. Within this broader context, our specific objectives were to (1) explore patient and public preferences regarding the initial evaluation of four anonymized, real‐world candidate drugs included in the AD‐SMART trial; (2) examine how individuals’ trade‐off between key medications attributes, such as side effects and dosage regimen, and to (3) assess whether and how these preferences vary across subgroups with different demographic backgrounds.

Given the scale of AD‐SMART (approximately 400 patients per arm) and the critical importance of patient and public involvement, this represented an ideal opportunity to pilot and evaluate a novel web‐based approach to determine whether it would be feasible to obtain involvement and engagement at scale.

**RESEARCH IN CONTEXT**

1. **Systematic review**: We searched Web of Science, PubMed, Embase, and EBSCO using terms related to “Patient and public involvement and engagement (PPIE)”, “dementia”, and “clinical trials”. Our review shows that most PPIE in dementia research have involved small, local groups, which restricts diversity and limits the generalizability of findings. Additionally, existing studies have sometimes focused narrowly on dissemination rather involving collaborators in a more integrated and participatory role. While online platforms have been used in other fields to expand engagement, evidence on whether web‐based approaches can deliver scalable PPIE in dementia research and capture public preferences for clinical trials remain scarce.
2. **Interpretation**: We investigated the potential for engaging with patients and the public at scale using a novel web‐based portal (POrtal for Patient and Public Engagement in Dementia [POPPED]) to facilitate mass feedback. Using the POPPED, we conducted a multilingual survey to explore public preferences on candidate drugs and public's views on a forthcoming Alzheimer's disease platform trial (AD‐SMART). More than 3000 people worldwide provided preferences on candidate drugs and attributes shaping drug preferences. Our findings show that side effects and efficacy are prioritized over dosing or monitoring, and that patterns are broadly consistent across demographic groups. These findings extend existing evidence by showing that digital platforms can complement traditional small scale PPIE, addressing its common limitations in reach and representativeness. Moreover, scaling PPIE survey activity enabled us to move beyond simply collecting opinions, allowing meaningful inference about interaction effects and providing insights into design of future dementia clinical trials.
3. **Future directions**: Future research should examine how to interpret and balance insights when small qualitative groups and large‐scale PPIE surveys yield inconsistent results, such as disagreements about which drugs to prioritize. It will also be important to refine the use of digital platforms, including extending discrete choice experiments to additional contexts and building on the established 2000+ cohort. Finally, efforts to reduce digital exclusion are needed to ensure that web‐based PPIE approaches are inclusive and complementary to traditional methods.

## 2 METHODS

### 2.1 Study settings

The broader context for this study is the AD‐SMART trial, an adaptive platform trial designed to evaluate multiple treatments for a single disease with the ability to add or remove treatments being tested over time dependent on interim results.^13^ Members of the wider team have successfully delivered such platforms in oncology and neurodegenerative diseases.^14^, ^15^, ^16^ The development of a systematic, sustainable drug prioritization pipeline is described in detail elsewhere.^17^ Briefly, this process involved engaging the wider AD community and inviting proposals for candidate treatments using a pre‐specified template, followed by expert panel meetings. Artificial intelligence (AI) guided reviews of the available literature were then used to create extended drug profiles for shortlisted drugs before further expert panel reviews with individualized rankings. Alongside this, a PPIE group provided qualitative feedback on the shortlisted drugs, based on lay summaries produced by the drug prioritization team. An overview of the relationship between AD‐SMART, POPPED and this survey, and PPIE activities is provided in Figure S1.

### 2.2 POPPED and the online survey

While existing PPIE activities, such as consultation with PPIE groups and expert review, were taken into account in the drug selection process, we also aimed to capture perspectives from a broader range of individuals who had not previously been invited to contribute these decisions. To achieve this, we designed the POPPED (https://popped.org.uk/)^18^ to host a range of projects and gather wider PPIE inputs on dementia research. The study protocol was approved by the research ethics committee of the University of Cambridge (REC Reference: HBREC.2025.01). All data was administered using the Research Electronic Data Capture (REDCap) platform and database securely hosted by the University of Cambridge.

We used the POPPED to examine attitudes to drugs being considered for inclusion in the AD‐SMART trial and the impact of characteristics of hypothetical medicines on whether people might be willing to take them in a trial. Specifically, the survey consisted of three main sections: (1) socio‐demographic background and their attitudes toward clinical trials that test more than one drug simultaneously against a placebo, that is, enthusiasm for a platform trial design, (2) a ranking task assessing participants’ overall preferences for four anonymized, real‐world Alzheimer's drugs with potential efficacy, and (3) a series of discrete choice experiments (DCEs) to explore trade‐offs among specific treatment attributes. The full survey is available at https://popped.org.uk/ad‐smart‐survey/.^19^ Data collection was carried out between March 7, 2025 and July 15, 2025 responses (see Figure S2 for more details).

**FIGURE 1:**
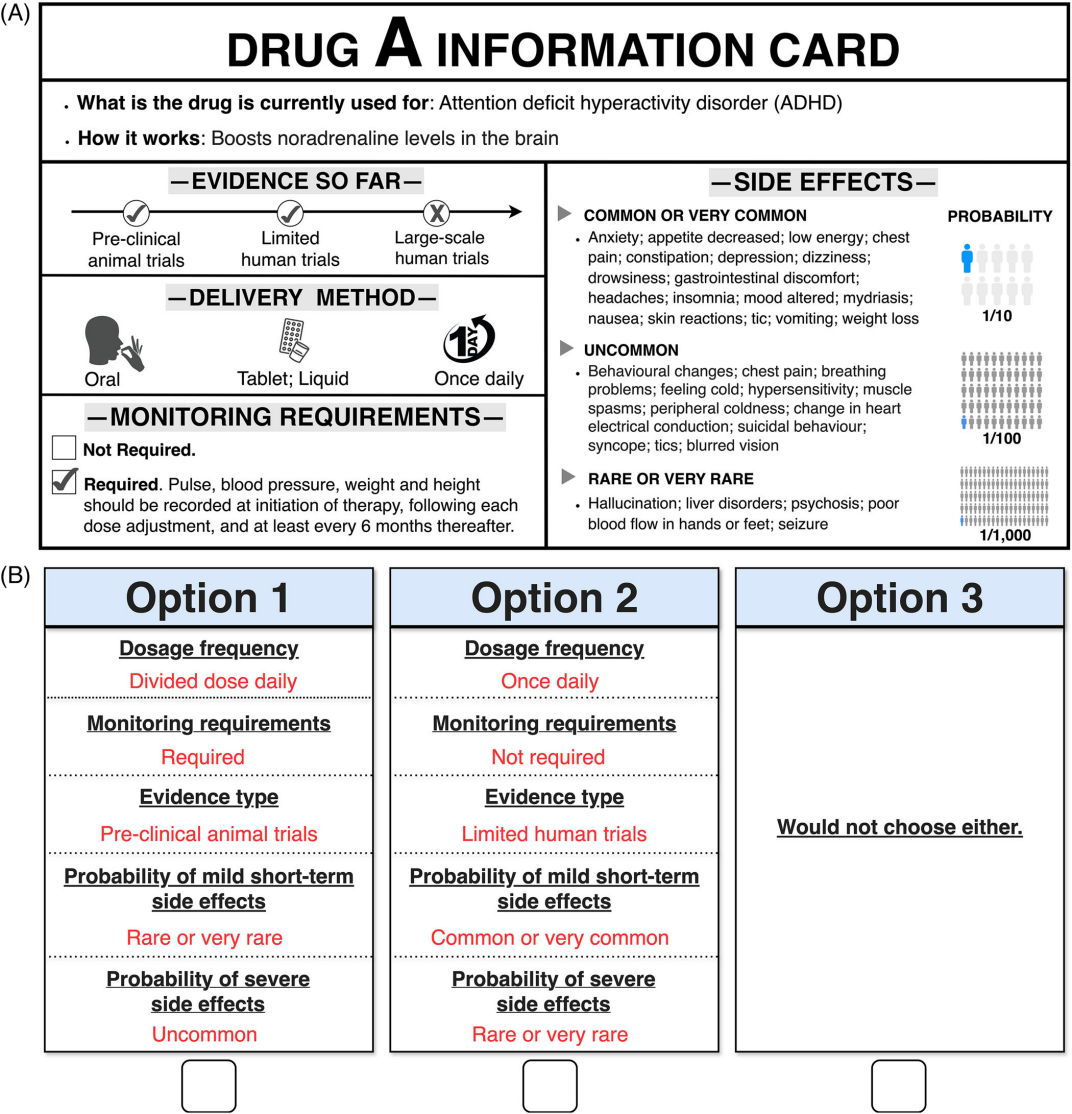
Materials used in the survey. (A) An example of the anonymized drug card. (B) An example of Choice task in DCEs.

Eligible participants for the Web‐based survey were individuals aged 18 years or older. All participants provided electronic consent prior to participation. Information to aid drug selection consisted of two main parts. First, in the ranking task, participants were presented with four anonymized drug cards representing the leading medications currently under consideration for inclusion in the trial. Based on the drug profiles, each card presented information on what the drug is currently used for, its proposed mechanism of action, the stage of clinical research or use, its delivery method, any special monitoring requirements, and its known side effects. Figure 1(A) provides an example of a drug card, and all drug cards are detailed in Figure S3. Participants were asked to rank these four drugs according to their overall preferences to inform prioritization discussions for the trial.

Participants then evaluated hypothetical drugs as opposed to actual treatments using a series of DCEs. These hypothetical profiles systematically varied across five key attributes identified in the preparatory PPIE sessions as being important: dosage frequency, monitoring requirements, type of evidence, probability of mild short‐term side effects, and probability of severe side effects. A detailed description of the attributes and levels is provided in Table S1.

Based on all possible combinations of attribute levels, 72 pairwise choice scenarios could be constructed. We used a D‐efficient experimental design to reduce respondents’ burden while maintaining statistical efficiency. This type of design minimizes the determinant of the covariance matrix of the parameter estimates, thereby maximizing the information derived from the selected choice sets.^20^ Consequently, 12 tasks were required to achieve reliable estimation. To further minimize respondent fatigue, a block design was employed in which 12 tasks were divided into four subsets. Each participant was randomly assigned to complete three choice tasks plus one additional attention check, resulting in four tasks in total (see Table S2). Figure 1(B) provides an example of a DCE choice task where participants chose between two hypothetical medications or selected neither option.

### 2.3 PPIE activities

PPIE was integrated throughout the preparatory stages of the online portal. Prior to the launch of the Web‐based survey, a series of PPIE meetings were conducted with a group of 14 individuals with lived experience of dementia, who had previously been involved in the AD‐SMART drug prioritization process. These contributors helped co‐develop key elements of the study, including the structure and language of the web‐based survey and the design of the online portal. They advised on the clarity, user‐friendly language, and accessibility of the materials, with particular attention to how uncertainty and risk were communicated. Participants also provided feedback on the overall cognitive and emotional burden of completing the survey, leading to several adjustments to improve flow, comprehension, and usability. Importantly, people shared their views on each of the shortlisted drugs, including which they found more or less appealing and why. These in‐depth qualitative insights helped inform the selection of attributes and levels used in the forthcoming survey, ensuring that the design was grounded in real‐world perceptions and concerns expressed by people with lived experience.

In terms of engagement strategy, we used multiple channels to reach diverse audiences. These included continued collaboration with the original PPIE groups, outreach via social media platforms (LinkedIn, Facebook, X, and WeChat), and promotion through the University of Cambridge, National Health Service (NHS) Trust, dementia research charities, and National Institute for Health and Care Research (NIHR) communications teams. The survey was also promoted on British Broadcasting Corporation (BBC) and commercial radio and TV interviews, which contributed to a steady but slow increase in responses. By far the largest boost in recruitment came from placing the survey on the Join Dementia Research (JDR) platform, which resulted in approximately 2000 responses (see Figure S2 for more details). To improve accessibility, the survey was made available in both English and Mandarin Chinese. These two languages were selected because English is the most widely spoken language in the world, while Mandarin Chinese has the largest number of native speakers.

The engagement was inclusive of a broad range of voices, including people living with dementia, family carers, professionals working in dementia, and other members of the public with relevant perspectives. Participation was entirely voluntary and no incentives were provided. These efforts aimed to ensure the accessibility and relevance of the study, and to support a more representative model of PPIE in research.

### 2.4 Statistical analysis

For the main analysis, we adopted a conservative power calculation approach, which required a minimum of approximately 500 participants to achieve adequate statistical power. As subgroup analyses were also planned, a substantially larger sample size was anticipated. Using the largest stratification by age (eight groups) as a reference, we targeted roughly 300 respondents per group, giving a total sample size of around 2900 participants after allowing for anticipated exclusions (see Supplementary S1 for sample calculation details). However, for subgroup analyses, the threshold (300) is highly conservative and often impractical in DCEs. Therefore, we set a pragmatic target of around 200 participants per subgroup to balance feasibility and statistical robustness. Overall, most subgroups achieved the intended sample size target, except for the subgroup of participants living with dementia (*n* = 90). Analyses for this subgroup were therefore considered exploratory.

For the ranking task, raw ranking results were visualized using heatmaps to display the frequency of each drug being ranked in each position. Weighted preference scores were then calculated using a scoring scheme (rank 1 = 4 points, rank 2 = 3, rank 3 = 2, rank 4 = 1). The total weighted score for each drug was calculated as:

$$\mathrm{Weighted}\;\mathrm{score}=\left(\eta_{1}*4\right)+\left(\eta_{2}*3\right)+\left(\eta_{3}*2\right)+\left(\eta_{4}*1\right)$$

where $\eta_{1}$—$\eta_{4}$ are the number of times the drug was ranked 1st through 4th. Bar charts were used to present the total weighted scores.

For the DCE, a mixed logit model was used to quantify respondents’ preferences for medication attributes and to account for preference heterogeneity across individuals. The utility $U_{ijt}$ of respondent i from choosing medication j in choice set t is specified as:

$$\begin{array}{ccc} U_{ijt} & = & \left(\beta_{1}+\eta_{1i}\right)\;\mathrm{Frequenc}y_{jt}+\left(\beta_{2}+\eta_{2i}\right)\;\mathrm{Monitorin}g_{jt}\\ & & +\left(\beta_{3}+\eta_{3i}\right)\;\mathrm{Evidenc}e_{jt}+\left(\beta_{4}+\eta_{4i}\right)\;\mathrm{Mil}d_{jt}\\ & & +\left(\beta_{5}+\eta_{5i}\right)\;\mathrm{Sever}e_{jt}+\varepsilon_{ijt} \end{array}$$

where $\mathrm{Frequenc}y_{jt},\;\;\mathrm{Monitorin}g_{jt},\;\;\mathrm{Evidenc}e_{jt},\;\;\mathrm{Mil}d_{jt},\;\;\mathrm{Sever}e_{jt}$ are dummy‐coded attribute levels. $\beta_{1\;}$ to $\beta_{5\;}$ are the mean utility weights for attributes k relative to their reference levels, while $\eta_{ki}$ capture the individual‐specific random deviations. The $\varepsilon_{ijt}$ is assumed to follow a Type I extreme value distribution.

Relative importance for each attribute was calculated based on the range of its estimated level coefficients, following the method described by Malhotra and Birks.^21^ The importance weight for attribute k was determined by:

$$\mathrm{Relative}\;\mathrm{Importance}\;=\frac{\mathrm{Range}\;\mathrm{of}\;\mathrm{coefficients}\;\mathrm{for}\;\mathrm{the}\;\mathrm{attribute}}{\sum \mathrm{Range}\;\mathrm{of}\;\mathrm{coefficients}\;\mathrm{for}\;\mathrm{all}\;\mathrm{attributes}}$$

We then conducted two subgroup analyses to explore whether preferences for medication attributes differed across population subgroups. The first analysis stratified participants by age and gender (female and male). The second analysis stratified them by dementia‐related experience. For each subgroup, we estimated separate models and between‐group comparisons. All analyses were conducted using Stata.

To examine whether the collected sample size was sufficient for stable parameter estimation, we also conducted a post hoc stability check by estimating the model with progressively larger subsamples (e.g., the first 500, 1000, 1500, and all respondents. See Supplementary S2). The results suggested that the key parameter estimates stabilized after approximately 1000–1500 respondents, supporting the adequacy of the final sample.

## 3 RESULTS

### 3.1 Sample characteristics

A total of 3251 respondents were included in the analysis. Responses were received from 27 countries across 6 continents, with the top five being the United Kingdom, China, the United States, Ireland, and Australia. The sample was primarily based on United Kingdom (87.4%), with a further 11.5% from China. A world map showing countries with survey responses is available in Figure S4.

As shown in Table 1, the largest age group was those aged 65–74 years, accounting for 31.28% of the sample. Overall, 71.2% of the total sample were aged 55 years and over, and 28.8% were aged 18–54 years. A majority identified as female (68.0%), while 31.3% identified as male. Most respondents indicated that their friends or family members had/have dementia (46.4%), followed by those who care for or have cared for someone with dementia (16.7%). Among those who reported having dementia, the majority (75.5%) were aged between 65 and 84 years, with 31.1% in the 65–74 age group and 44.4% in the 75–84 age group. Only a small proportion were younger than 55 years (10.0%) or aged 85 and over (3.3%).

**TABLE 1:** Participants characteristic.

Characteristics	Frequency (Percentage) *N* = 3251
Age, years	
18–24	175 (5.4%)
25–34	283 (8.7%)
35–44	171 (5.3%)
45–54	306 (9.4%)
55–64	808 (24.9%)
65–74	1017 (31.3%)
75–84	453 (13.9%)
85 and over	38 (1.2%)
Gender	
Female	2209 (68.0%)
Male	1018 (31.3%)
Another gender	14 (0.4%)
Prefer not to answer	10 (0.3%)
Participants’ description	
My friend or family member had/has dementia	1507 (46.4%)
I care/have cared for someone with dementia	544 (16.7%)
My work is related to dementia	182 (5.6%)
I have dementia	90 (2.8%)
None of the above	883 (27.2%)
Prefer not to say	45 (1.4%)
Residence region	
United Kingdom	2842 (87.4%)
China	375 (11.5%)
United States	5 (0.2%)
Ireland	4 (0.1%)
Australia	3 (0.1%)
Others	22 (0.7%)

Overall, our sample demonstrated broad demographic and experiential diversity, supporting the robustness and potential external relevance of the study findings across different age groups, genders, personal experiences with dementia, and geographic contexts.

### 3.2 Preferences ranking results

#### 3.2.1 Overall preferences

**FIGURE 2:**
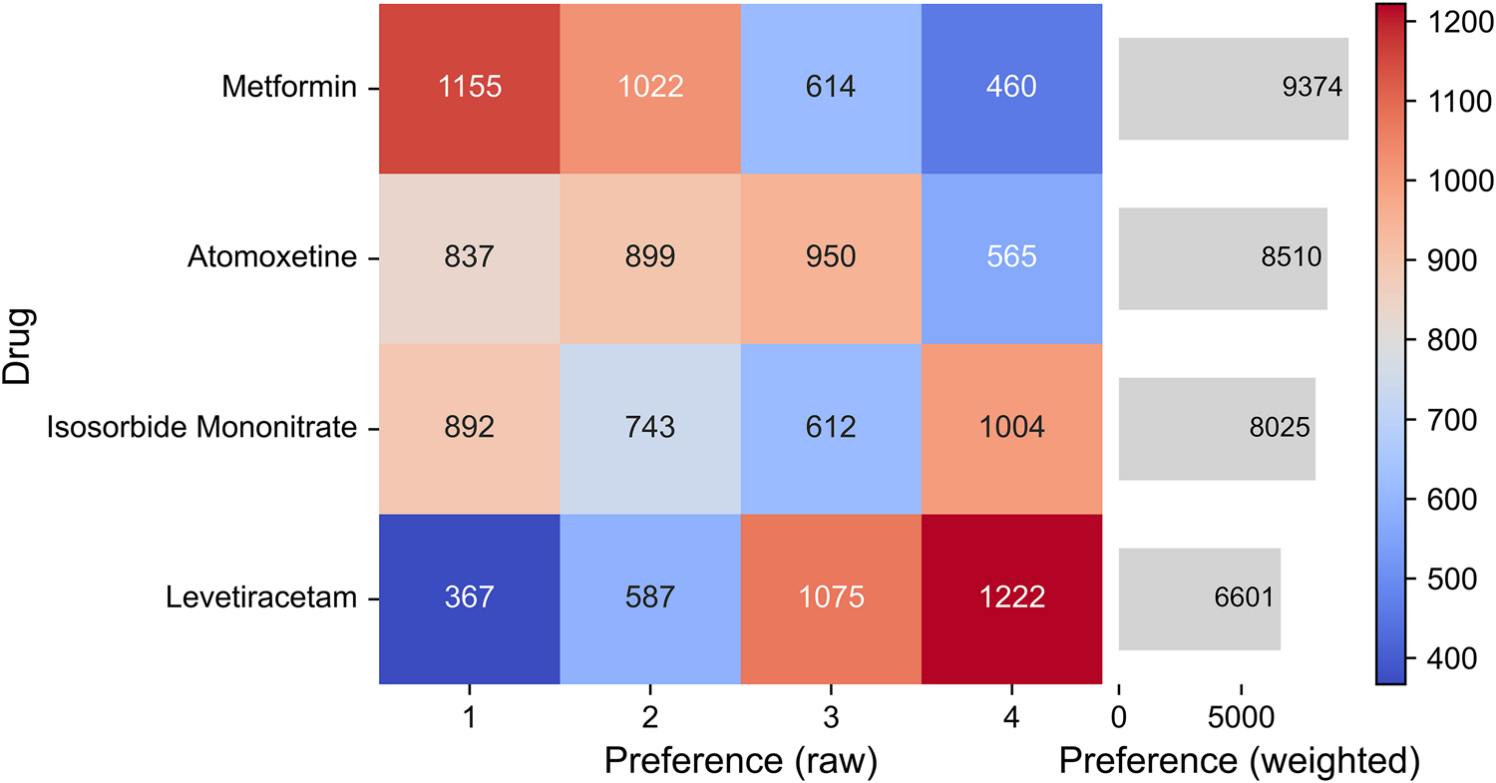
Preferences for four drugs across ranking positions. The heatmap (left) displays the raw counts of each drug ranked 1st to 4th by participants. Darker red indicates stronger preference, while darker blue indicates weaker preference. The bar chart (right) shows the total weighted preference scores for each drug.

Figure 2 shows the distribution of participants’ preferences for each drug. The heatmap on the left shows that metformin was most frequently selected as the top choice (1st rank: n = 1155, 35.5%), followed by isosorbide mononitrate (n = 892, 27.4%), atomoxetine (n = 837, 25.7%), and levetiracetam (n = 367, 11.3%). Levetiracetam was most commonly chosen as the fourth preference (*n* = 1,222, 37.6%), indicating a tendency for it to be ranked lower compared to other options.

The bar chart on the right summarizes the weighted preference scores. The results indicate that metformin accumulated the highest total weighted counts at the top rank (total weighted count = 9374), followed by atomoxetine, isosorbide mononitrate, and levetiracetam. Notably, even after weighting, metformin remained predominantly favored as a first‐line option, while levetiracetam was more often considered suitable as a last choice.

#### 3.2.2 Subgroup preference ranking

**FIGURE 3:**
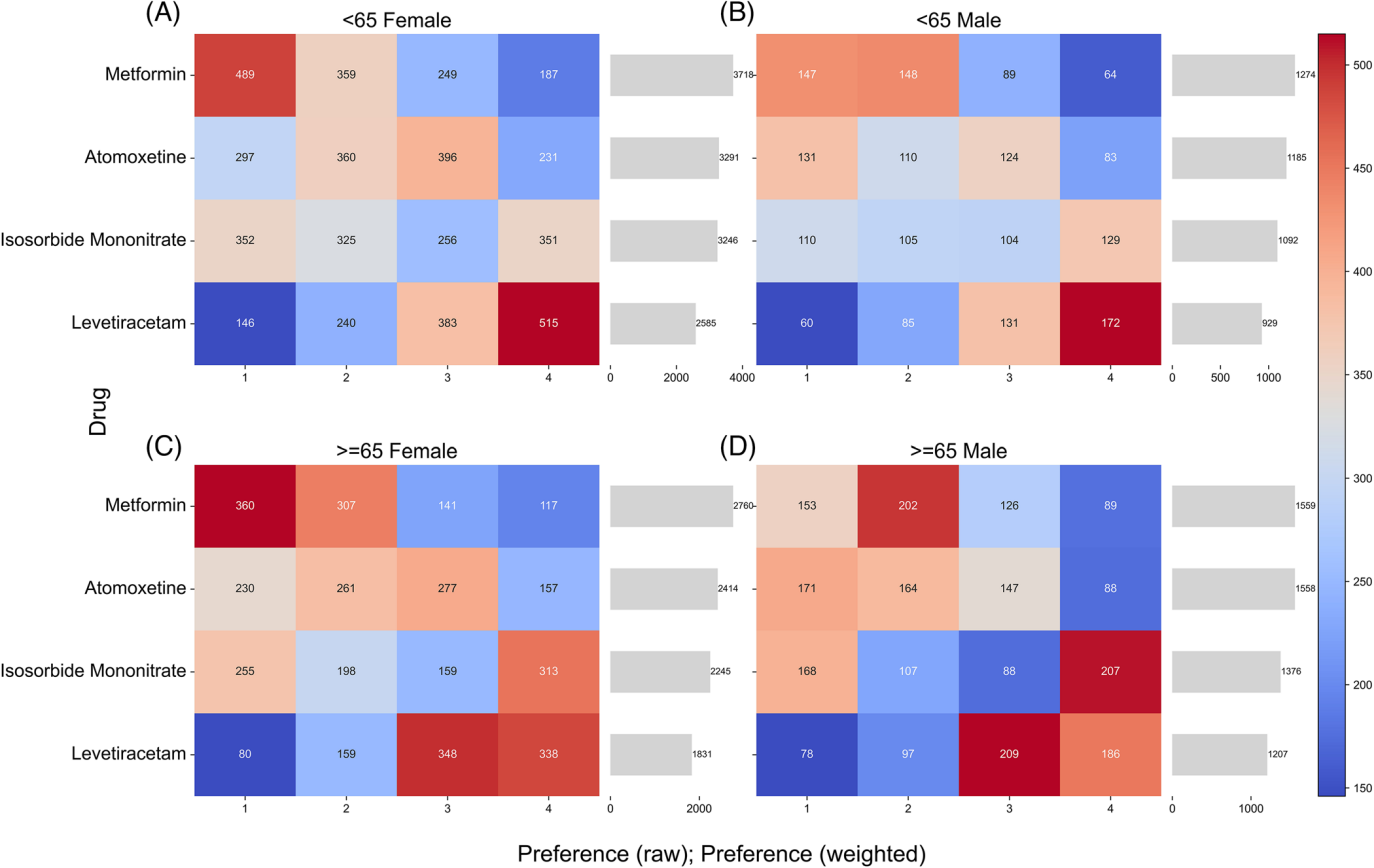
Preferences by age and gender. The heatmaps show the distribution of raw ranking frequencies for each drug. Darker red indicates stronger preference, while darker blue indicates weaker preference. The bar charts show the corresponding total weighted preference scores. Results are presented separately for four participant groups: (A) < 65 Female, (B) < 65 Male, (C) ≥ 65 Female, and (D) ≥ 65 Male.

*Age and gender*: To further explore potential demographic heterogeneity in drug preferences, we stratified the sample by age and gender. Although eight age categories were used at the data collection stage, analyzing them in combination with gender would create too many small subgroups with limited sample size. Therefore, we combined them into broader categories using 65 years as the cutoff. The threshold of 65 years was selected for two reasons. First, our data showed that the majority of respondents with dementia (78.8%) were aged over 65, indicating a concentration of samples in this age range. Second, 65 years old is a widely used clinical cutoff for young‐onset dementia or working‐age dementia.^22^, ^23^ Figure 3 presents results for each subgroup.

Across all four subgroups, the ranking pattern was consistent with that of the full sample: metformin received the highest preference, followed by atomoxetine, isosorbide mononitrate, and levetiracetam. The weighted preference scores also suggest similar preference structures.

**FIGURE 4:**
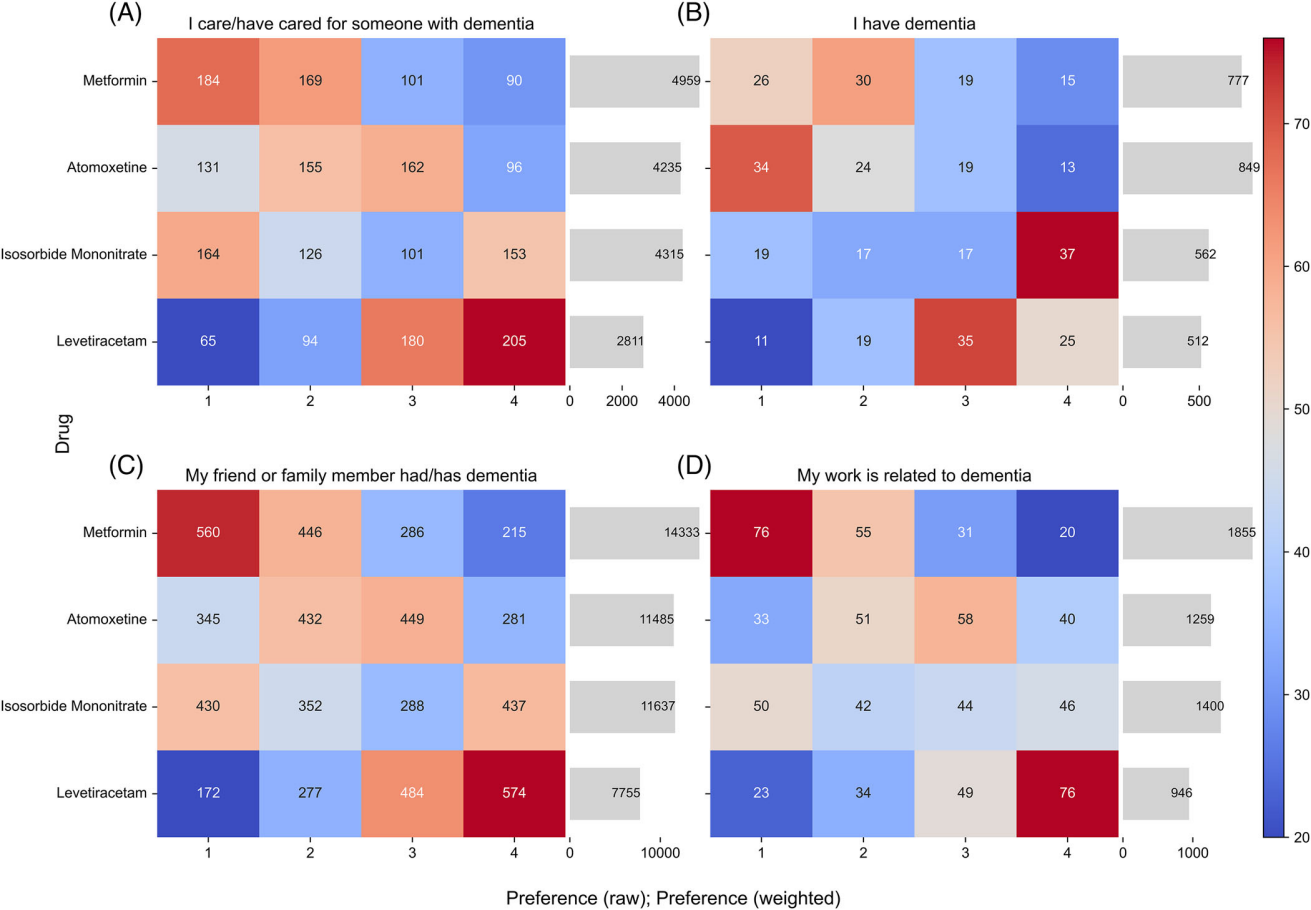
Preferences across participants’ role. The heatmaps show the distribution of raw ranking frequencies for each drug. Darker red indicates stronger preference, while darker blue indicates weaker preference. The bar charts show the corresponding total weighted preference scores. Results are presented separately for four participant groups: (A) caregivers, (B) individuals with dementia, (C) friends or family of people with dementia, and (D) professionals working in dementia‐related fields.

*Dementia‐related experience*: In addition to demographic characteristics, we further explored whether people's experience with dementia influences their preferences. Figure 4 presents rankings across participant subgroups based on their experience with dementia. Each panel displays raw preference distributions and the corresponding weighted scores for the four drugs.

Across the subgroups, two distinct patterns emerged. Participants who had caregiving experience (a), had a friend or family member with dementia (c), or worked in dementia‐related fields (d) showed similar preference structures, where metformin was consistently the most preferred drug, followed by isosorbide mononitrate and atomoxetine, and levetiracetam was the least favored. In contrast, participants living with dementia themselves (b) showed a different pattern, with atomoxetine slightly surpassing metformin, and the other two drugs receiving the lowest scores.

### 3.3 DCE results

#### 3.3.1 Overall results

**FIGURE 5:**
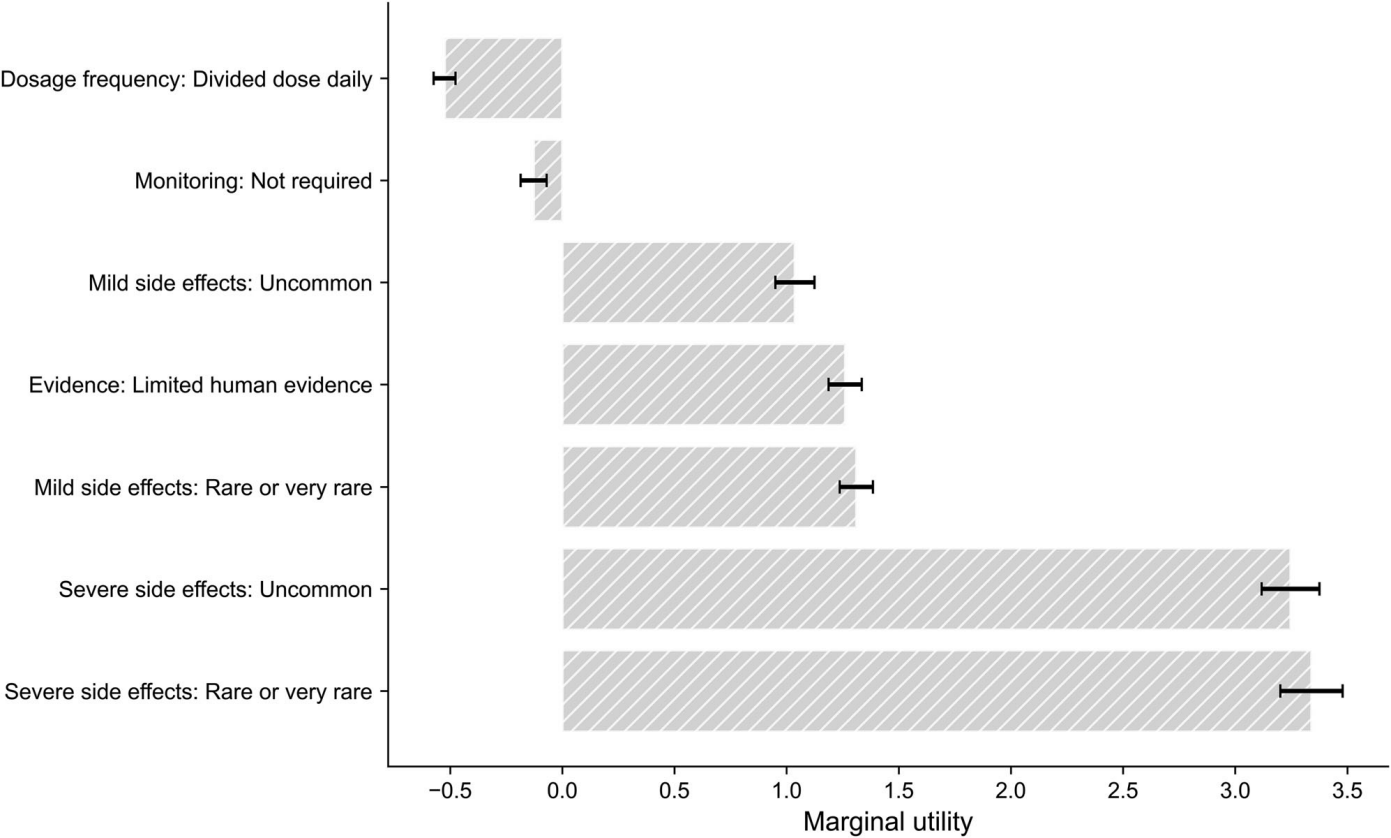
Marginal utilities of attribute levels. Error bars represent the estimated marginal utility for each attribute level relative to its reference level. Reference levels were: Dosage frequency: once daily; Monitoring: required; Evidence: pre‐clinical animal trials; Mild side effects: common or very common; Severe side effects: common or very common. Error bars denote 95% CI.

We estimated a mixed logit model to examine public preferences across five treatment attributes (see Table S3). Relative importance of each attribute was calculated based on the range of their estimated coefficients. Figure 5 displays the marginal utilities of the attribute levels.

All five attributes had a statistically significant effect on participants’ choice. Among these, severe side effects exhibited the highest impact on decision‐making (relative importance: 50.88%). Both levels of severe side effect (Uncommon and Rare or very rare) showed strong positive marginal utilities (β=3.247 and 3.340, respectively; both p<0.001), indicating that respondents placed the greatest weight on avoiding severe adverse outcomes when evaluating medications. Similarly, mild side effects were found to be influential (relative importance: 19.97%), with significant preferences for medications associated with less frequent mild side effects (β=1.037 for Uncommon and β=1.311 for Rare or very rare, both p<0.001), suggesting sensitivity to the probability of experiencing mild discomfort.

The third most important factor was the type of evidence (relative importance: 19.21%). Specifically, respondents preferred medications supported by stronger human evidence over those based solely on pre‐clinical animal data (β=1.261, p<0.001).

In contrast, dosage frequency (relative importance: 8.0%) and monitoring requirements (relative importance: 1.95%) had relatively small effects. Divided daily dosing (β=−0.525, p<0.001) and not requiring monitoring (β=−0.128, p=0.029) were both associated with lower utility.

#### 3.3.2 Subgroup analyses

**FIGURE 6:**
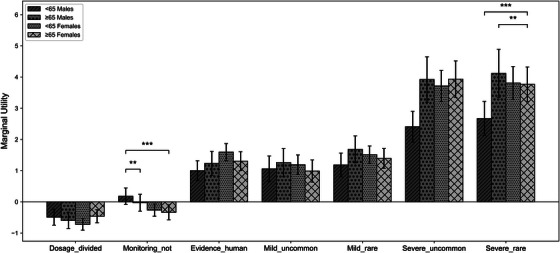
Subgroup marginal utilities with 95% CI. Bars represent four demographic groups (< 65 males, ≥65 males, < 65 females, ≥65 females). Significant pairwise differences are indicated by asterisks (∗p<0.05,∗∗p<0.01,∗∗∗p<0.001).

*Age and gender*: Figure 6 presents subgroup analyses stratified by age and gender, including four groups: males aged < 65 years, males aged ≥ 65 years, females aged < 65 years, and females aged ≥ 65 years.

Across all groups, attributes related to side effects (both Uncommon or Rare) remained the strongest positive influence on treatment choice, followed by the type of evidence, whereas dosage frequency and monitoring requirements contributed less.

Notably, for the monitoring requirement, both male subgroups (< 65 and ≥65 years old) did not exhibit statistically significant preferences. In contrast, both female subgroups demonstrated significant preferences for medications involving regular monitoring. The group comparison tests further revealed a significant difference between younger and older males. Specifically, younger males (< 65 years old) showed a greater preference for drugs that do not require monitoring, whereas older males (≥ 65 years old) tended to prefer those that involve regular monitoring (p<0.01). This finding suggests a potential age‐related divide among males in terms of their preference for ongoing health monitoring. In addition, we also observed a significant difference between younger males and older females (p<0.001). Younger males (< 65 years old) were relatively more inclined to prefer drugs without frequent monitoring, while older females (≥ 65 years old) were comparatively more favorable towards regular health monitoring.

In addition, the magnitude of preferences for severe side effects varied by age and gender. Younger males (< 65 years old) and older females (≥ 65 years old) differed significantly in their preferences, with older females showing a stronger aversion to severe side effects (p<0.001). Additionally, we observed a significant difference between older males and older females (≥ 65 years). The results showed that men in the older age group place even more weight on avoiding severe side effects compared to their female counterparts (p<0.01).

**FIGURE 7:**
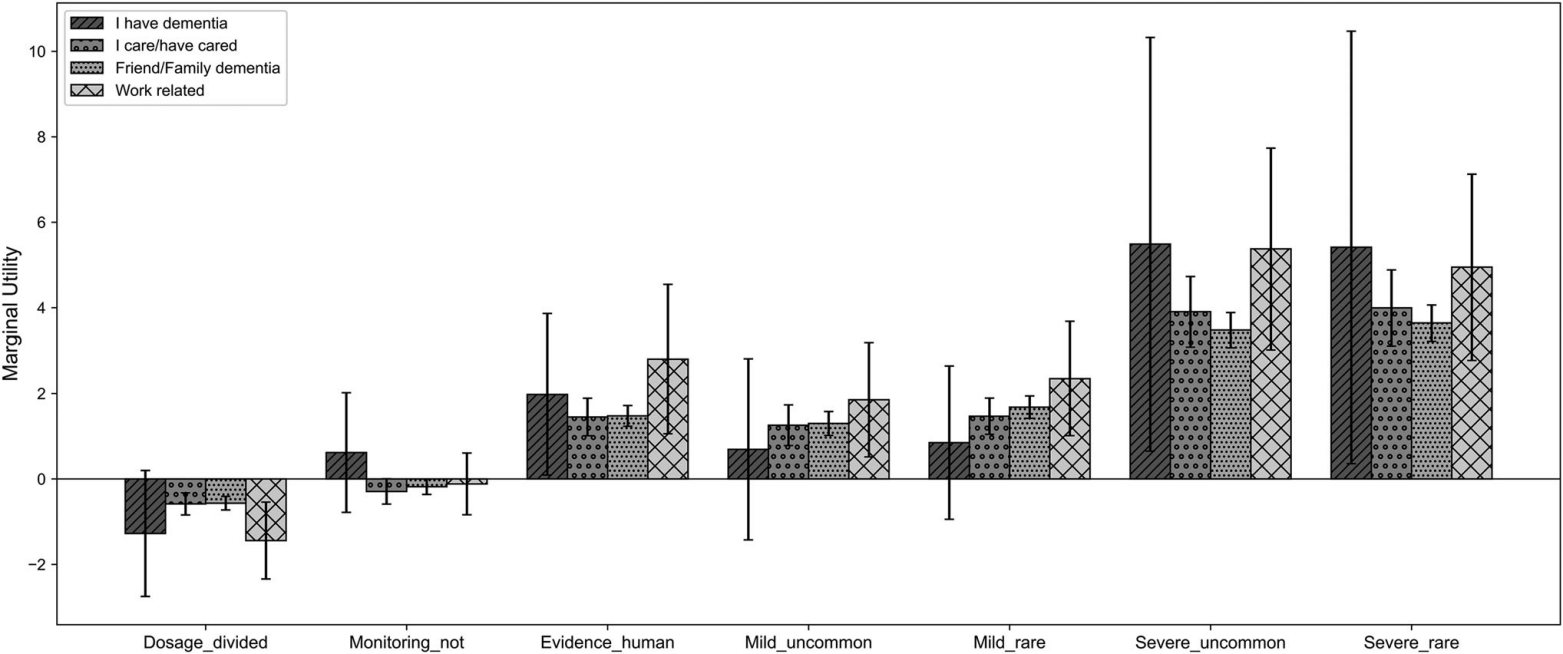
Subgroup marginal utilities with 95% CI. Bars represent four groups with different dementia experience. Significant pairwise differences are indicated by asterisks (∗p<0.05,∗∗p<0.01,∗∗∗p<0.001).

*Dementia‐related experience*: Figure 7 further illustrates the estimated marginal utilities and 95% CI for groups with different dementia experience: I have dementia, I care/have cared for someone with dementia, my friend or family member had/has dementia, and my work is related to dementia.

The overall preference patterns across dementia‐related experience were broadly consistent with those observed in the full sample. Side effects remained the strongest determinants, followed by type of evidence, whereas dosage frequency and monitoring requirements contributed minimally.

Although no statistically significant differences were detected between subgroups, an exploratory finding was observed for the group living with dementia. For these participants, only severe side effects and type of evidence had a statistically significant positive influence on treatment preferences, while mild side effects, dosage frequency and monitoring requirements did not significantly affect their decision‐making.

## 4 DISCUSSION

### 4.1 Summary

We have demonstrated it is possible to use a web‐based portal to engage with patients and the public at scale to help design a research project in dementia. We were also able to understand views on our research approach, obtain rankings for four compounds for repurposing in Alzheimer's Disease and, crucially, understand factors that are most important in making these decisions. We found that people were enthusiastic about the idea of a platform trial in AD, with 79.6% of participants reporting very positive or somewhat positive attitudes.

Among the four candidate drugs, they favored *Metformin*, followed by *Atomoxetine* and *Isosorbide Mononitrate* and were less enthusiastic about *Levetiracetam*. These findings will be used within the AD‐SMART platform trial as part of a wider PPIE contribution to the drug selection process. Specifically, the online survey complemented more focused PPIE meetings in which each candidate drug was discussed in depth, alongside expert reviews. Together, these informed the prioritization of candidate drugs, with *Metformin* and *Atomoxetine* being identified as prioritized choices.

The DCE findings indicate that the main driver of decision making was avoiding serious side effects, followed by mild side effects and type of evidence, as well as dosage frequency and monitoring requirements. This is consistent with previous studies showing that patients and the public prioritize medication side effects as the most important factors when making decisions about treatment.^24^ However, the interpretation of monitoring is complex and vary across subgroups. Our subgroup analyses revealed notable gender and age‐related differences in preferences for monitoring. Female participants showed a stronger preference for regular monitoring, while male participants overall did not exhibit significant preference. The reasons may be complex. One possible explanation is that women are generally more engaged in health maintenance, and they may perceive monitoring as a source of reassurance. ^25^, ^26^ In addition, regular monitoring and check‐ups may also be experienced as a form of empowerment, enabling them to feel more actively involved in their care and better able to manage potential risks.^27^ The group comparison tests also highlighted an age‐related divide among male participants. Men under 65 years were relatively more inclined to prefer drugs without monitoring, whereas men over 65 years tended to favor those involving regular monitoring. This pattern may reflect differences in health priorities and risk perceptions: Younger men may perceive themselves to be at lower health risks, while older men typically have a higher risk perception and are more willing to undergo frequent monitoring for age‐related conditions.^26^, ^28^

### 4.2 Strengths and limitations

The major strength of our study is its large and diverse sample, eliciting the views of more than 3000 people from all over the world. Our digital platform complements existing small scale PPIE addressing its common limitations in reach and representativeness. This approach enabled broader engagement by removing geographic and logistical barriers, offering multilingual access, and creating opportunities for large numbers of individuals to express their views. Moreover, scaling PPIE survey activity enabled us to go beyond simply capturing opinions, allowing meaningful inference about interaction effects and providing insights into the factors that make certain medications more or less attractive to take. Feedback on the survey experience also indicated that the vast majority of participants found it easy to complete, with only around 10% reporting any difficulty. This suggests that the survey was broadly accessible and that the co‐design input from PPIE contributors helped ensure usability.

Another important strength lies in the cost‐effectiveness of this approach. Once established, our platform allowed large‐scale engagement at minimal additional cost. Participation was entirely voluntary and with no financial incentives offered, yet engagement remained high. This demonstrates strong public willingness to contribute to dementia research when the process is accessible and meaningful, reinforcing the value of web‐based PPIE as a sustainable model.

Finally, more than half of the respondents agreed to be contacted again, establishing a unique cohort of nearly 2000 individuals who can be directly reached in future PPIE activities. POPPED is a resource available for future studies, at least four are already planning to use it.

Our study does have some limitations. First, as an anonymous online survey, there is an inherent risk of fraudulent or erroneous responses. Online recruitment also requires internet access and a degree of digital literacy, raising the possibility of digital exclusion. For those who are less comfortable with technology, including some older adults or people with limited access to digital devices, may therefore have been underrepresented. In our study, the digital exclusion may also partly account for the relatively low participation of people with dementia (*n* = 90). Whilst this number is small, hearing the views of 90 people with dementia is important and it is also helpful to be able to identify their views separately to carers or others.

Second, the representativeness of the sample may be limited. Regionally, the sample was primarily base in the UK (87.4%) and very limited representation from other countries. Linguistically, the survey was only available in English and Mandarin Chinese, excluding those who do not speak either language. In addition, the small size of some subgroups, particularly individuals living with dementia (*n* = 90), which is below the target specified in the power analyses, may limit the validity and generalizability of these subgroup findings. The recruitment sources may introduce bias. A substantial proportion of respondents were recruited via JDR, where the volunteers may have greater background knowledge and more positive attitudes towards dementia research than the general public. Therefore, the views captured here may not fully reflect those of the wider population.

Finally, while the survey questions were carefully designed, they cannot fully capture the complexity of participants’ reasoning and may have influenced responses through the framing of choices. For example, attributes such as monitoring requirements may be interpreted in different ways, being seen as either an added burden or a source of reassurance. Future studies should complement survey findings with qualitative approaches and PPIE discussion to gain deeper insights into how such attributes are understood.

### 4.3 Future study

Our study represents a complementary approach aimed at expanding the reach and inclusivity of public input. We hope others will use POPPED and it will become an important and useful tool for PPIE in dementia research. The project has also raised new questions for future investigation. For instance, in our study, findings from the small qualitative group were broadly consistent with those from the large‐scale online survey. However, it remains unclear how researchers should interpret results when these approaches diverge, for example, if there were disagreements about which drugs to include. How should we weigh a rich qualitative discussion with a small number of people against an impersonal survey with a much larger sample size? Determining how to balance these complementary sources of evidence needs further exploration. While the ideal way of taking PPIE into account is yet to be determined, the feasibility of incorporating large‐scale public preferences into the decision‐making process will continue to be refined in future rounds of AD‐SMART.

In addition, we were pleased to be able to demonstrate that we could use the platform to obtain additional insights on factors that influenced drug preference through the use of DCEs. The portal highlights its potential to address many more questions in the future. Future work should also focus on understanding and minimizing digital exclusion, while acknowledging that this portal is designed to complement others approaches to PPIE. Lastly, although in the present study we limited translations to English and Chinese Mandarin due to time and financial constraints, future work should extend this multilingual approach to include other widely spoken languages such as Hindi, Spanish, and Arabic to reach broader populations.

In summary, we present what we believe to be the largest PPIE exercise conducted to support a single study in Alzheimer's disease. It will provide insights which will directly inform a forthcoming major trial platform and is a sustainable infrastructure which could be used by many future studies, improving their quality and strengthening recruitment, retention, and the generalizability of findings through better alignment with participant needs and preferences.

## CONFLICT OF INTEREST STATEMENT

The authors declare no conflicts of interest.

## CONSENT STATEMENT

All participants in the survey provide electronic consent prior to participation.

## Supporting information



Supporting Information

Supporting Information

## APPENDIX 1 FULL LIST OF AD‐SMART CONTRIBUTORS

### 1.1

AD‐SMART Core Team

Professor Paresh Malhotra, Imperial College London—p.malhotra@imperial.ac.uk

Professor James Carpenter, MRC Clinical Trials Unit, University College London—j.carpenter@ucl.ac.uk

Professor Vanessa Raymont, University of Oxford—vanessa.raymont@psych.ox.ac.uk

Professor Ross Dunne, Greater Manchester Mental Health NHS Foundation Trust/University of Manchester—ross.dunne@gmmh.nhs.uk

Professor Suzanne Reeves, UCL—suzanne.reeves@ucl.ac.uk

Dr Benjamin R Underwood, University of Cambridge – bru20@cam.ac.uk

Linda Pointon, University of Cambridge—ljp22@medschl.cam.ac.uk

Shabinah Ali, MRC Clinical Trials Unit—shabinah.ali@ucl.ac.uk

Dr Sabahat Iqbal, Imperial College London ‐ s.iqbal@imperial.ac.uk

Cristina Bonet‐Olivares, Imperial College London—c.bonet-olivares23@imperial.ac.uk

Jo Whittle, MRC Clinical Trials Unit, University College London – joanne.whittle@ucl.ac.uk

Laura Rizzo, Imperial College London—l.rizzo1@imperial.ac.uk

**Wider Team for Appendix**

Fleur Hudson, MRC Clinical Trials Unit—f.hudson@ucl.ac.uk

Isobel Landray, LSHTM—isobel.landray1@lshtm.ac.uk

Suzie Cro, Imperial College London—s.cro@imperial.ac.uk

Helen Brooks, UK DRI—helen.brooks@ukdri.ac.uk

Dr Caroline S Clarke, UCL—caroline.clarke@ucl.ac.uk

Lindsey Masters, MRC Clinical Trials Unit—l.masters@ucl.ac.uk

Sara Peres, MRC Clinical Trials Unit—sara.peres@ucl.ac.uk

Carlos Diaz Montana, MRC Clinical Trials Unit—c.diaz@ucl.ac.uk

Professor Max Parmar, MRC Clinical Trials Unit – m.parmar@ucl.ac.uk

Professor Jeremy Chataway, MRC Clinical Trials Unit/UCL Queen Square—j.chataway@ucl.ac.uk

Professor Siddharthan Chandran, UK DRI—siddharthan.chandran@ed.ac.uk

Professor Suvankar Pal, University of Edinburgh—suvankar.pal@ed.ac.uk

Professor Henrik Zetterberg, UK DRI—h.zetterberg@ucl.ac.uk

Professor Dave Sharp, Imperial College London—david.sharp@imperial.ac.uk

Professor Nick Fox—n.fox@ucl.ac.uk

Professor Liz Coulthard, University of Bristol—Elizabeth.Coulthard@bristol.ac.uk

Dr Amanda Heslegrave, UK DRI—a.heslegrave@ucl.ac.uk

Professor Dame Louise Robinson, Newcastle University—a.l.robinson@newcastle.ac.uk

Professor Clive Ballard, University of Exeter—c.ballard@exeter.ac.uk

Dr Timothy Rittman, University of Cambridge—tr332@medschl.cam.ac.uk

Dr Daniel Blackburn, University of Sheffield—d.blackburn@sheffield.ac.uk

Professor Dag Aarsland, Kings College London—dag.aarsland@kcl.ac.uk

Professor Alan Thomas, Newcastle University—alan.thomas@newcastle.ac.uk

Professor Masud Husain, University of Oxford—masud.husain@ndcn.ox.ac.uk

Professor James Rowe, University of Cambridge—james.rowe@mrc-cbu.cam.ac.uk

Professor Charles Marshall, Queen Mary University of London—charles.marshall@qmul.ac.uk

Dr Ivan Koychev, University of Oxford—ivan.koychev@psych.ox.ac.uk

Dr Jay Amin, University of Southampton—jay.amin@soton.ac.uk

Dr Tom Russ, University of Edinburgh—T.C.Russ@ed.ac.uk

Dr Simon Bell, University of Sheffield—s.m.bell@sheffield.ac.uk

Dr Jonathan Blackman, University of Bristol—jonathan.blackman@bristol.ac.uk

Dr Robert Barber, University of Newcastle—Robert.Barber@cntw.nhs.uk

Dr Tomas Welsh, University of Bristol—tomas.welsh@nhs.net

Dr Zunera Khan, the Care Network zunera.2.khan@kcl.ac.uk

Professor John O'Brien—john.obrien@medschl.cam.ac.uk

**Drug prioritisation panel – independent members**

Professor Jeffrey Cummings—jcummings@cnsinnovations.com

Professor Fiona Ducotterd—f.ducotterd@ucl.ac.uk

Professor Eric Karran—eric.karran@abbvie.com

Professor John O'Brien—john.obrien@medschl.cam.ac.uk

Professor Alastair Reith—alastair.d.reith@outlook.com

Professor Lon Schneider—lschneid@usc.edu

Professor Rik Vandenberghe—rik.vandenberghe@uzleuven.be

Professor Gordon Wilcock—gordon.wilcock@retired.ox.ac.uk

Professor Cath Mummery—c.mummery@ucl.ac.uk

**Independent PPIE members**:

Ajay Dave

Eric Deeson

Brian Human

Jakki Levene

Pamela Marie Lumbroso

Remy Olasoji

Heather Richardson

Ian Richardson

Martin Robertson

David Ross

Charlotte Scrimgeour

Sheila Wonnacott

David Winskill

Winnie Henry

Charity Representatives

Dr Zunera Khan, the Care Network zunera.2.khan@kcl.ac.uk

Amy Kordiak, Alzheimer's Society Amy.Kordiak@alzheimers.org.uk

Emma Wolverson, Dementia UK Emma.Wolverson@dementiauk.org

Anna Goodman, ARUK anna.goodman@alzheimersresearchuk.org

Dr Leah Mursaleen leah.mursaleen@alzheimersresearchuk.org

Dr Sheona Scales Sheona.Scales@alzheimersresearchuk.org

Dr Josie Waters
