# Supplementary material for: Patient and public involvement and engagement in clinical trials at scale: Analysis of the first 3250 responses on the POrtal for Patient and Public Engagement in Dementia (POPPED)
Source: Alzheimers Dement. 2026 Feb 28;22(2):e71113. doi: 10.1002/alz.71113 (PMC12949453; doi:10.1002/alz.71113)
Supplement: Supplementary file 1 — Supporting Information [file ALZ-22-e71113-s001.docx]

# Supplementary Materials


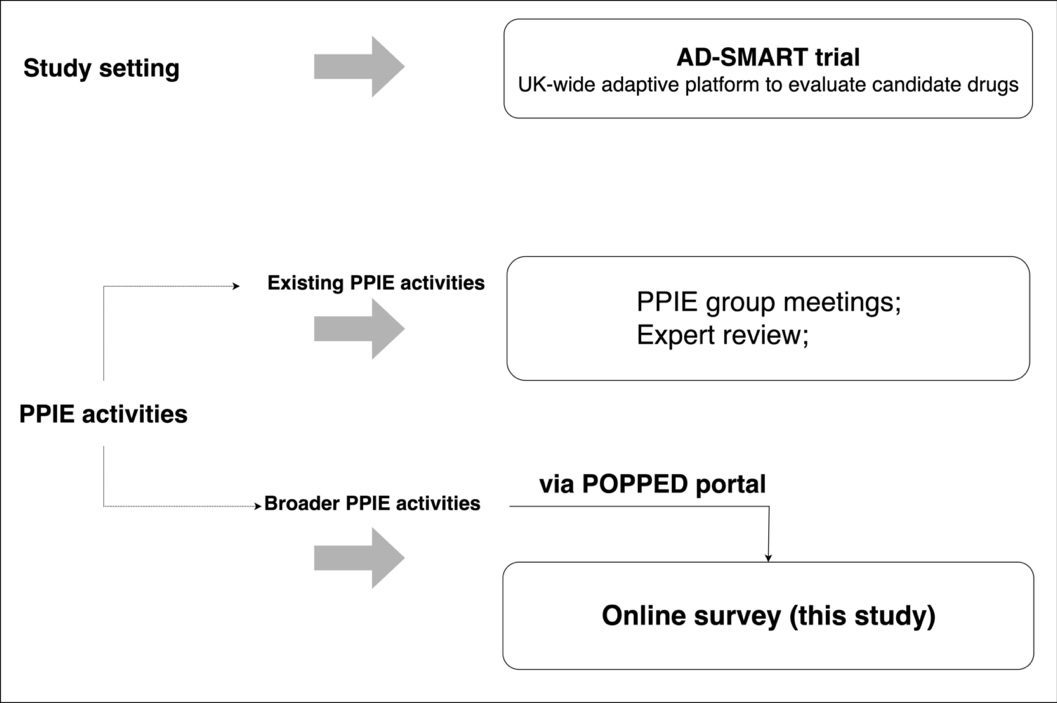


## FIGURE S1 Relationship between AD-SMART, POPPED and this survey, and PPIE activities.

The setting for this study was AD-SMART (Alzheimer’s Disease–Systematic Multi-Arm Adaptive Randomised Trial), a UK-wide clinical trial platform designed to evaluate candidate drugs for Alzheimer’s disease. A key question for AD-SMART is which candidate drugs should be prioritised for initial evaluation, and this requires input from patients and the public. While traditional PPIE activities, such as consultation with PPIE groups and expert review, were taken into account in the drug selection process, we also aimed to capture perspectives from a broader range of individuals who had not previously been invited to contribute to these decisions. To achieve this, we developed the POPPED (POrtal for Patient and Public Engagement in Dementia), an online platform designed to host a range of PPIE studies in dementia research. Within this context, the present online survey was conducted to gather public views on candidate drugs and to explore factors that may influence preferences.


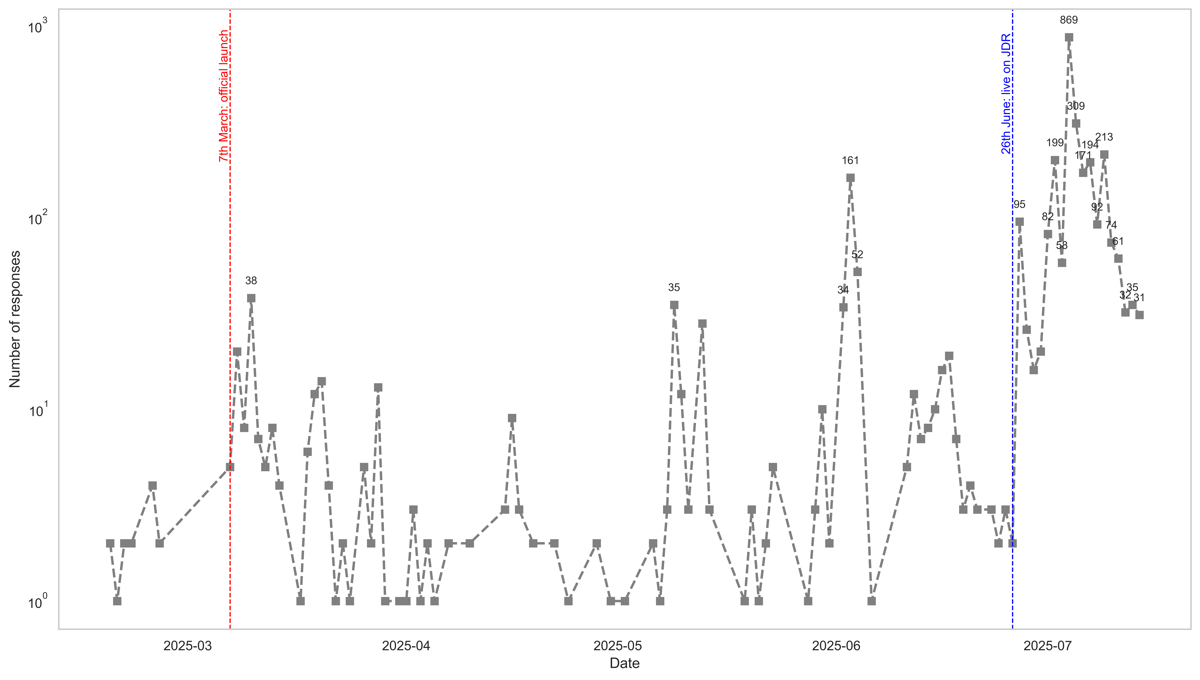


## FIGURE S2 Survey responses over time. Daily number of survey completions (logarithmic scale) from March to July 2025. The red dashed line marks the launch of the survey website, and the blue dashed line marks the study’s appearance on the Join Dementia Research (JDR) platform on 26 June 2025.


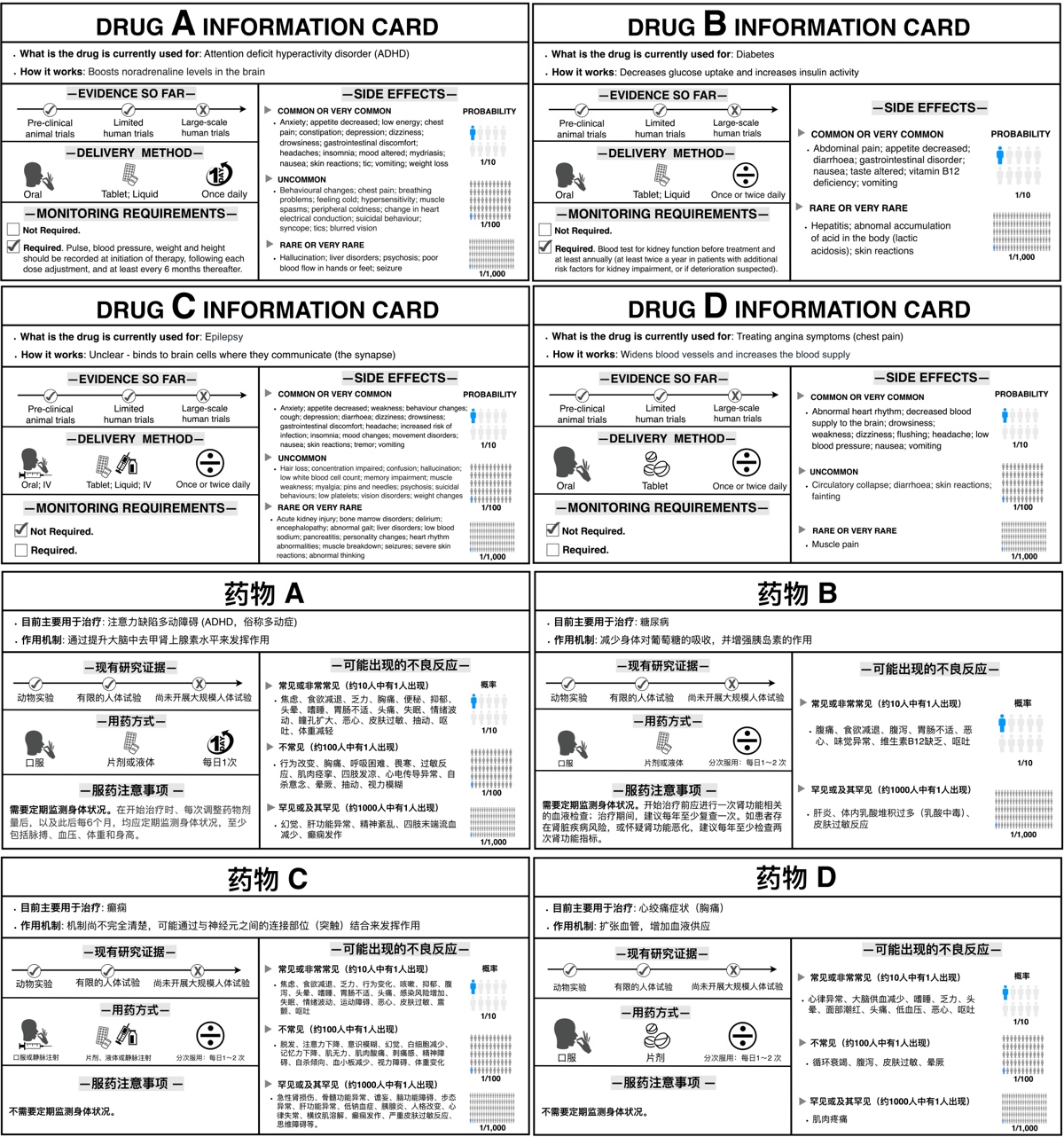


## FIGURE S3 Detailed drug cards in English and Mandarin Chinese.


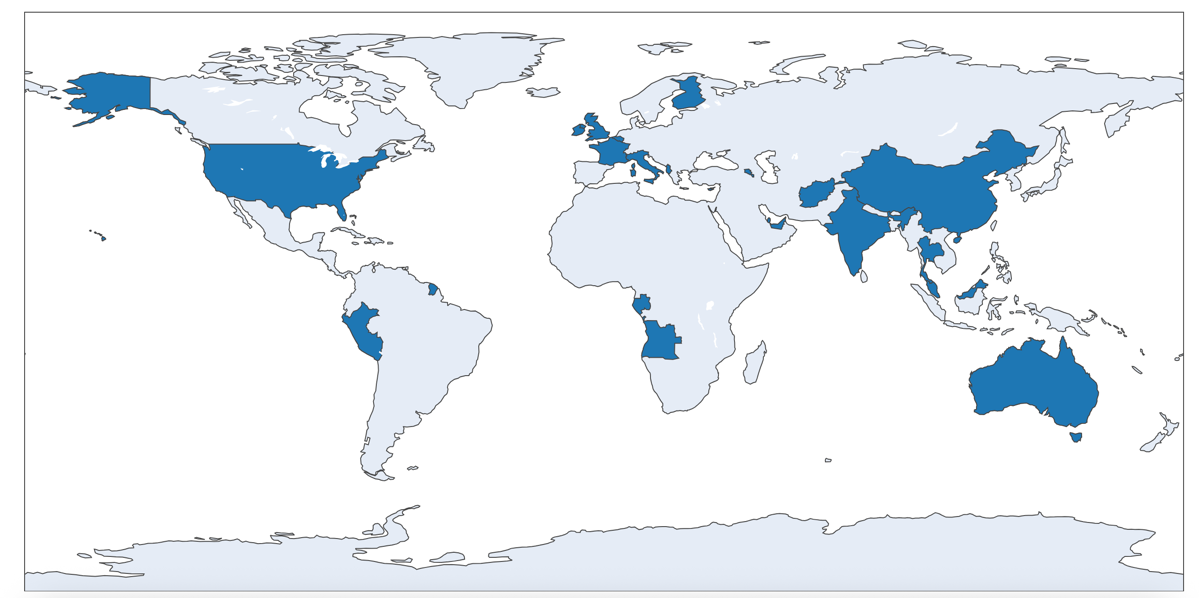


## FIGURE S4 Geographic distribution of respondents (darker blue = countries represented)

## TABLE S1 Attributes and levels in the DCE element.

| **Attributes** | **Definition** | **Levels of attributes** | **Description** |
| --- | --- | --- | --- |
| Dosage frequency | The amount of medication taken at a specific time. | Once daily | Only need to be taken once a day. |
|  |  | Divided dose daily | The total dose should be divided into several doses per day. |
| Monitoring requirements | Whether people need medical testing and monitoring before and after taking the drug. | Required | People are required to undergo medical tests and monitoring, such as liver function tests, kidney function tests, and blood tests, before and after using the medication. |
|  |  | Not required | People are not required to undergo additional medical tests before and after using the medication. |
| Evidence type | The type or stages of research conducted, ranging from pre-clinical studies to limited human evidence. | Pre-clinical animal evidence | Evidence comes solely from animal models or in vitro studies, with no human trials yet. |
|  |  | Limited human evidence | Small-scale or early-phase human studies suggest potential benefits but lack robust results. |
| Probability of mild short-term side effects | These include common side effects that are generally mild and may resolve on their own within a short period. These may include: dizziness, nausea, diarrhea, and headaches. | Very common and common | 1 out of 10 people |
|  |  | Uncommon | 1 out of 100 people |
|  |  | Rare or very rare | 1 out of 1,000 people |
| Probability of severe side effects | Severe side effects that may necessitate medical intervention or hospitalization. These may include severe palpitations, nephropathy, lactic acidosis, reversible renal impairment, respiratory disorders, fainting, severe allergic reactions, cardiac arrhythmias, and severe hepatic dysfunction. | Very common and common | 1 out of 10 people |
|  |  | Uncommon | 1 out of 100 people |
|  |  | Rare or very rare | 1 out of 1,000 people |

## TABLE S2 Sixteen choice tasks selected in the experimental design.

| **Block** | **Dosage frequency** | **Monitoring requirement** | **Efficacy evidence** | **Mild side effects** | **Severe side effects** |
| --- | --- | --- | --- | --- | --- |
| 1 | Divided dose daily | Required | Pre-clinical animal trials | Rare or very rare | Uncommon |
|  | Once daily | Not required | Limited human trials | Common or very common | Rare or very rare |
|  | Divided dose daily | Not required | Pre-clinical animal trials | Rare or very rare | Rare or very rare |
|  | Once daily | Required | Limited human trials | Uncommon | Common or very common |
|  | Once daily | Required | Pre-clinical animal trials | Common or very common | Uncommon |
|  | Divided dose daily | Not required | Limited human trials | Uncommon | Common or very common |
|  | Divided dose daily | Required | Pre-clinical animal trials | Rare or very rare | Uncommon |
|  | Once daily | Not required | Limited human trials | Common or very common | Rare or very rare |
| 2 | Once daily | Required | Limited human trials | Rare or very rare | Common or very common |
|  | Divided dose daily | Not required | Pre-clinical animal trials | Uncommon | Uncommon |
|  | Once daily | Required | Limited human trials | Rare or very rare | Uncommon |
|  | Divided dose daily | Not required | Pre-clinical animal trials | Uncommon | Common or very common |
|  | Divided dose daily | Required | Limited human trials | Common or very common | Uncommon |
|  | Once daily | Not required | Pre-clinical animal trials | Rare or very rare | Rare or very rare |
|  | Once daily | Required | Limited human trials | Rare or very rare | Common or very common |
|  | Divided dose daily | Not required | Pre-clinical animal trials | Uncommon | Uncommon |
| 3 | Once daily | Not required | Pre-clinical animal trials | Uncommon | Uncommon |
|  | Divided dose daily | Required | Limited human trials | Common or very common | Common or very common |
|  | Divided dose daily | Not required | Limited human trials | Common or very common | Rare or very rare |
|  | Once daily | Required | Pre-clinical animal trials | Uncommon | Common or very common |
|  | Once daily | Required | Pre-clinical animal trials | Common or very common | Rare or very rare |
|  | Divided dose daily | Not required | Limited human trials | Rare or very rare | Uncommon |
|  | Once daily | Not required | Pre-clinical animal trials | Uncommon | Uncommon |
|  | Divided dose daily | Required | Limited human trials | Common or very common | Common or very common |
| 4 | Divided dose daily | Required | Pre-clinical animal trials | Rare or very rare | Rare or very rare |
|  | Once daily | Not required | Limited human trials | Common or very common | Uncommon |
|  | Divided dose daily | Required | Limited human trials | Uncommon | Rare or very rare |
|  | Once daily | Not required | Pre-clinical animal trials | Rare or very rare | Common or very common |
|  | Once daily | Required | Limited human trials | Uncommon | Rare or very rare |
|  | Divided dose daily | Not required | Pre-clinical animal trials | Common or very common | Common or very common |
|  | Divided dose daily | Required | Pre-clinical animal trials | Rare or very rare | Rare or very rare |
|  | Once daily | Not required | Limited human trials | Common or very common | Uncommon |

Note: Based on drug characteristics and level, there were 72 hypothetical medications and 2,556 possible choice tasks between two hypothetical drugs. A D-efficient experimental design was created with STATA software to reduce the number of choice tasks to twelve. In addition, a repeated choice set was used to check the consistency of respondents’ choices.

## TABLE S3 Mixed logit model result (full sample).

| Mixed logit model | | |  | Number of obs = 23,056 | | |
| --- | --- | --- | --- | --- | --- | --- |
|  | |  |  | LR chi2(7) = 1052.95 | | |
| Log likelihood = -5738.6682 | | |  | Prob > chi2 = 0.0000 | | |
|  |  |  |  |  |  |  |
| chosen | Coef. | Std. Err. | z | P>\|z\| | [95% Conf.Interval] | |
| **Mean** |  |  |  |  |  |  |
| dosage_divided | -0.525 | 0.049 | -10.670 | **0.000** | -0.622 | -0.429 |
| monitoring_not | -0.128 | 0.058 | -2.190 | **0.029** | -0.242 | -0.013 |
| evidence_human | 1.261 | 0.074 | 17.130 | **0.000** | 1.116 | 1.405 |
| mild_uncommon | 1.037 | 0.087 | 11.890 | **0.000** | 0.866 | 1.208 |
| mild_rare | 1.311 | 0.074 | 17.620 | **0.000** | 1.165 | 1.457 |
| severe_uncommon | 3.247 | 0.129 | 25.230 | **0.000** | 2.995 | 3.500 |
| severe_rare | 3.340 | 0.139 | 24.030 | **0.000** | 3.068 | 3.613 |
| **SD** |  |  |  |  |  |  |
| dosage_divided | 0.663 | 0.131 | 5.070 | **0.000** | 0.407 | 0.919 |
| monitoring_not | 1.828 | 0.097 | 18.840 | **0.000** | 1.637 | 2.018 |
| evidence_human | 1.641 | 0.122 | 13.460 | **0.000** | 1.402 | 1.880 |
| mild_uncommon | 1.803 | 0.283 | 6.380 | **0.000** | 1.249 | 2.357 |
| mild_rare | 0.409 | 0.199 | 2.050 | **0.040** | 0.019 | 0.800 |
| severe_uncommon | 0.202 | 0.284 | 0.710 | 0.476 | -0.355 | 0.759 |
| severe_rare | 0.766 | 0.320 | 2.390 | **0.017** | 0.139 | 1.393 |

Note: all categorical variables were dummy-coded.

Dosage: Once daily (baseline)

Monitoring: Required (baseline)

Evidence: Pre-clinical animal trials (baseline)

Mild side effects: Common or very common (baseline)

Severe side effects: Common or very common (baseline)

A positive coefficient indicates a higher preference compared to the baseline level.

## S.1 Study sample

The minimum sample size required for the DCE was determined using a standard parametric approach for choice probability estimation:

$$n \geq\frac{(1-p)}{rpa^{2}}\times\left( \Phi^{-1}\left( 1 -\frac{\alpha}{2} \right) \right)^{2}$$

where $p$ is the expected true population probability (set to 0*.*5 for maximum variance), $r$ is the number of choice tasks completed per respondent (*3* in our design), $a$ is the acceptable margin of error around the true population probability (0*.*05), and $\Phi^{-1}$ is the inverse of the cumulative normal distribution function, and $\alpha$ is the significance level ($\alpha$ = 0*.*05). Therefore, a minimum of approximately 500 participants was required to maintain overall statistical power.

However, because we planned to conduct subgroup analyses by gender, age, respondent role and country, we needed to ensure that each subgroup would have an adequate sample size for meaningful comparisons. In particular, we focused on the largest stratification by age, which included eight groups (18-24, 24-34, 35-44, 45-64, 55-64, 65-74, 75-84, 85 and over). By targeting roughly 200 respondents per age group to ensure sufficient precision for meaningful comparisons. Allowing for an anticipated 20% exclusion, the overall sample size was set at around 2,900 participants to ensure sufficient statistical power for subgroup analyses robust comparisons across key demographic and contextual factors.

## S.2 Model stability check

we conducted a post hoc model stability check to assess whether further data collection continued to improve the reliability of parameter estimates. Specifically, we re-estimated the model in stages, progressively increasing the number of respondents (e.g., 500, 1000, 1500, and 3000), and plotted the estimated coefficients for all attribute levels with 95% confidence intervals.


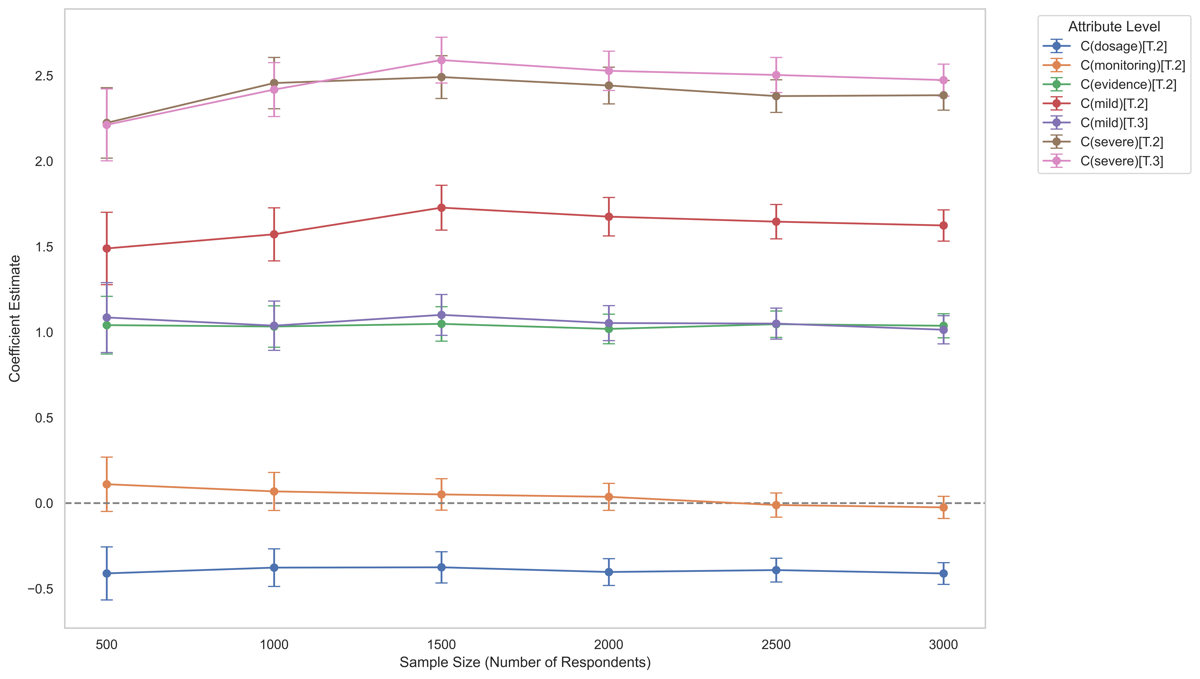


Figure S.2. Coefficient estimates by sample size

As shown in Figure S.2, most key coefficients stabilized after approximately 1000-1500 respondents, and confidence intervals narrowed with increasing sample size. This suggests that the data reached saturation well before the full sample size was collected, and additional responses had little impact on the model conclusions.
